# Local Housing Choice Voucher Distribution Policies Impact Healthcare Utilization: a Randomized Natural Experiment

**DOI:** 10.1007/s11524-022-00609-7

**Published:** 2022-03-16

**Authors:** Hannah Cohen-Cline, Kyle Jones, Keri B. Vartanian

**Affiliations:** Providence Health System: Providence Health and Services, Portland, USA

**Keywords:** Housing, Health Care Utilization, Public policy

## Abstract

While associations between obtaining affordable housing and improved health care are well documented, insufficient funding often forces housing authorities to prioritize limited housing vouchers to specific populations. We assessed the impact of obtaining housing on health care utilization at two urban housing authorities with different distribution policies: Housing Authority A prioritized seniors and people with disabilities, while Housing Authority B prioritized medically complex individuals and families with school-aged children. Both housing authorities used random selection to distribute vouchers, allowing us to conduct a randomized natural experiment of cases and waitlisted controls. No significant demographic differences were present between those receiving vouchers and waitlisted controls. Housing Authority A vouchers were associated with increased outpatient visits (OR = 1.19; *P* = 0.051). Housing Authority B vouchers decreased the likelihood of emergency department visits (OR = 0.61; *P* = 0.042). This study provides evidence that, while obtaining housing can result in better health care outcomes overall, local prioritization policies can influence that impact.

## Introduction

The USA is embroiled in a severe affordable housing crisis. Shortages in affordable housing and skyrocketing rents have caused nearly 11.4 million households to face severe rent burden, [1, 2] defined as spending more than 50% of their income on rent. Among households earning less than 30% of the area median income, over 60% are severely rent burdened [1]. This level of rent burden often leads to housing instability and forces individuals and families to cut back on critical expenses needed to meet basic needs, including health care [2].

The association between housing and health care is well documented. Prior studies have linked housing to increased connection with primary and outpatient care [3–5] and decreased use of emergency department and inpatient services[3–7], and reduced total health expenditures [3, 4, 8–10]. Until the affordable housing supply is able to keep up with the demand, this housing crisis will continue to adversely affect population health.

To help ameliorate this crisis, the U.S. Department of Housing and Urban Development (HUD) provides rental assistance to over 5 million low-income households [11]. The dominant rental assistance program for low-income individuals and families is HUD’s Sect. 8 Housing Choice Voucher (HCV) Program. Approximately half of all households receiving federal rent assistance use this program, which provides vouchers that can be used to subsidize housing in the private market provided the housing meets strict safety and sanitation codes [12]. Voucher recipients pay 30% of their adjusted gross monthly income on rent and the voucher provides the additional funds.

However, unlike entitlement programs in which anyone meeting eligibility requirements is legally entitled to assistance, not everyone who is eligible for housing assistance receives it because there are not enough housing assistance funds available. In fact, the majority of housing unstable and rent-burdened individuals and families in the USA receive no assistance from HUD [11]. Housing authorities are therefore forced to make decisions on how to distribute a limited number of vouchers to their population, and different housing authorities may make different decisions about how to prioritize their vouchers [13].

Despite the well-documented association between housing and health care, little is known about how these types of local housing policy decisions about voucher distribution impact population health. This study uses a natural experiment occurring in two geographically adjacent cities on the West Coast to understand the health impacts of their distinct voucher distribution policies. Housing Authority A prioritized vouchers to seniors/people with disabilities and individuals engaged in an education and employment training program. Housing Authority B in a neighboring city implemented a policy to only distribute vouchers to homeless medically complex individuals and homeless families with school-aged children. Both housing authorities used random selection to distribute vouchers; we took advantage of the random selection to conduct a randomized natural experiment exploring the impact of different prioritization decisions on the health care utilization patterns of individuals obtaining housing assistance through the HCV program.

## Methods

We conducted two natural experiments using random selection to assess the impact of obtaining housing with a HCV voucher on health care utilization outcomes in two geographically adjacent urban cities of similar size and demographics.

### Study Population

Our study population consisted of adult Medicaid enrollees 18 to 65 years of age seeking housing assistance through the HCV program at either housing authority. At Housing Authority A, approximately 3000 applicants were assigned a random order to be selected off the waitlist between 2014 and 2016. We defined our treatment group as individuals selected for a voucher before July 2015, and our control group as individuals remaining on the waitlist during this time. At Housing Authority B, approximately 800 households applied to the HCV program waitlist between 2015 and 2017. We defined our treatment group as individuals selected at random for a voucher from October 2015, when the new prioritization policy started, to February 2017, when voucher selection was paused due to funding concerns. Our control group consisted of individuals who remained on the waitlist during this time.

To ensure we had enough claims data to construct our health care outcomes, individuals were excluded if they were had less than 3 months of Medicaid enrollment during the study window. We also excluded applicants over 65 years of age as we did not have access to Medicare data.

### Data

We used administrative data from each housing agency, which included: dates for application, voucher selection, eligibility determination, voucher issue, and obtaining housing; and applicant demographic characteristics. We matched this to Medicaid enrollment and claims data using probabilistic matching on name and date of birth. Medicaid data contained eligibility spans and all diagnostic and procedure codes for all health care encounters. We used this data to construct the following categories of health care visits: emergency department, inpatient, outpatient, outpatient mental health, and dental.

### Analysis

We created binary measures of health care utilization (used/did not use) in the 12 months after an individual’s index date. For both treatment groups, the index date was the date at which the applicant was selected for a voucher. For the Housing Authority A control group, the index date was assigned 12 months prior to their eventual voucher selection date; for the Housing Authority B control group, the index date was the date they applied to the waitlist.

Not everyone selected for a voucher obtains housing; we therefore used an instrumental variable design to estimate the impact of obtaining housing through the HCV program on our health care outcomes while preserving the benefits of randomization [14].

We conducted a two-stage least-squares (2SLS) regression using voucher selection as our instrumental variable, obtaining housing with the voucher as our exposure, and the health care utilization measures as our outcomes. The first stage regression predicted housing status (housed/not housed) from voucher selection status; the second stage regression used predicted housing status to assess the impact of housing on health care utilization. The second stage model provides the main coefficient of interest, which is interpreted as the impact of obtaining housing on health care utilization among individuals who obtain housing through the HCV program. More details on this analysis are given in Appendix.

All analyses were conducted in R version 3.3.3 [15]. All study procedures were approved by either the Providence Health & Services Institutional Review Board (IRB) or the Washington State IRB.

## Results

**Fig. 1:**
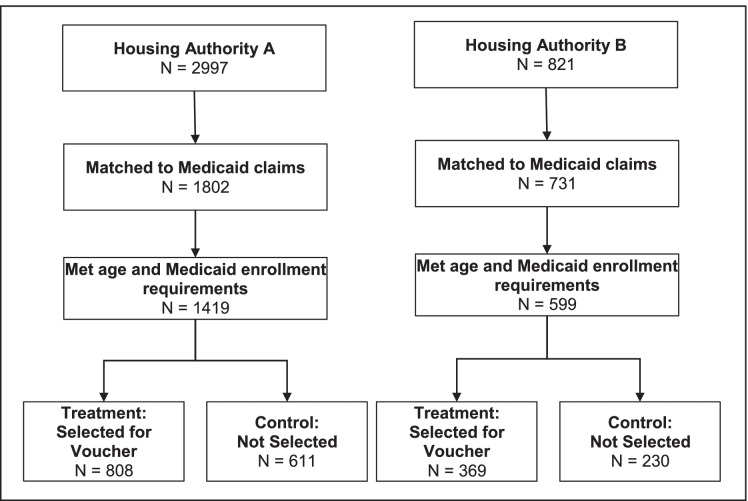
Study population and eligibility criteria for study participants at each public housing agency site

Figure 1 shows the final sample size for the analysis. Of the 2997 applicants to the Housing Authority A waitlist, 1802 (60%) matched to Medicaid data, and 1419 (47%) met the age and length of Medicaid enrollment criteria. A total of 808 applicants formed the treatment group, while 611 formed the control group. Of the 821 applicants on the Housing Authority B waitlist, we were able to match 731 to Medicaid data (89%), and 599 (73%) met the age and length of Medicaid enrollment criteria. A total of 396 applicants were selected to apply for a voucher, while 230 remained on the waitlist.

As expected due to the random nature of voucher selection, there were few substantial differences in demographics or medical complexity between the treatment and control groups at either site (Table 1). At Housing Authority A, those in the treatment group were slightly older than their control counterparts; while at Housing Authority B, those in the treatment group were slightly younger than their control counterparts.

**Table 1:** Study population demographics and health status

	Housing Authority A				Housing Authority B			
	Treatment *N* = 808		Control *N* = 611		Treatment *N* = 369		Control *N* = 230	
	*N*	%	*N*	%	*N*	%	*N*	%
Age at waitlist entry								
*18–25*	133	16.5%	114	18.7%	36	5.7%	15	6.5%
*26–35*	234	29.0%	178	29.1%	246	42.3%	90	39.1%
*36–45*	150	18.6%	126	20.6%	201	30.9%	87	37.8%
*46–55*	166	20.5%	137	22.4%	87	16.3%	27	11.7%
*56–65*	125	15.5%	56	9.2%	29	4.9%	11	4.8%
Sex								
*Female*	533	66.0%	397	65.0%	499	82.4%	195	84.8%
*Male*	275	34.0%	214	35.0%	100	17.6%	35	15.2%
Race								
*White*	416	51.5%	313	51.2%	484	81.0%	185	80.4%
*Black*	275	34.0%	202	33.1%	58	9.5%	23	10.0%
*Other*	117	14.5%	96	15.7%	57	9.5%	22	9.6%
Hispanic	153	18.9%	97	15.9%	61	11.4%	19	8.3%
CDPS^a^	1.44	1.37	1.35	1.35	1.40	1.36	1.25	1.39

^a^*CDPS*, Chronic Illness and Disability Payment System; mean and standard deviation

Among the Housing Authority A population, we also assessed the percent of applicants who were members of the priority population. There were no differences between the treatment and control group; approximately, a third of the population was seniors or people with disabilities and a third were participants in the education and employment training program (data not shown).

A total of 305 (38%) applicants from the treatment group at Housing Authority A obtained housing with their voucher. At Housing Authority B, 136 (37%) applicants from the treatment group obtained housing with their voucher.

Table 2 gives the percent of applicants who used each domain of health care at both sites and the results from the 2SLS regression model. The percentage of applicants using any given domain of health care at either site ranged from a little over 10% for inpatient stays to between 65 and 80% for outpatient visits. Almost half the sample population had at least one emergency department visit during the study window; by contrast, less than a third had visited a dentist.

**Table 2:** Impact of obtaining housing through the HCV program on health care utilization

	Control	Treatment	2SLS Model		
	%	%	RR	95% C.I	*p*-value
Housing Authority A					
Inpatient	10.64%	11.76%	1.23	0.55, 2.76	0.608
Emergency department	44.19%	43.19%	0.93	0.68, 1.26	0.630
Outpatient mental health	20.62%	21.91%	1.02	0.59, 1.74	0.953
Ambulatory outpatient	66.12%	72.65%	1.19	1.00, 1.43	0.051
Dental	22.42%	23.51%	1.36	0.85, 2.18	0.204
Housing Authority B					
Inpatient	11.74%	14.09%	1.40	0.43, 4.64	0.577
Emergency department	49.57%	42.01%	0.61	0.38, 0.98	0.042
Outpatient mental health	18.26%	22.22%	1.52	0.66, 3.54	0.327
Ambulatory outpatient	76.52%	79.40%	1.09	0.86, 1.38	0.471
Dental	27.39%	32.25%	1.55	0.78, 3.08	0.206

*2SLS*, two-stage least squares; *RR*, relative risk; *CI*, confidence interval. Housing Authority A analysis adjusted for age, CDPS score, time on the waitlist until study entry, sex, race, ethnicity, and membership in priority population. Housing Authority B analysis adjusted for age, CDPS score, time from the index date until study close, sex, race, and ethnicity

From the 2SLS regression model in the Housing Authority A population, obtaining housing through the HCV program was associated with a 1.19 greater risk of having at least one outpatient visit, but had no association with any of the other domains of health care. We also examined the type of outpatient care (primary care or specialty) and found that this increase was driven primarily by the use of specialty care (data not shown). From the 2SLS regression model in the Housing Authority B population, obtaining housing through the HCV program was associated with a nearly 40% lower risk of having at least one emergency department visit, but had no association with any of the other domains of health care.

## Discussion

This natural experiment provides evidence that obtaining housing through HUD’s HCV program impacts health care utilization. Applicants who obtained housing through the HCV program at Housing Authority A were more likely to have an outpatient visit, while those who obtained housing through the HCV program at Housing Authority B were less likely to use the emergency department, compared to their control counterparts. These results are aligned with previous research showing associations between housing instability and greater use of emergency department services [3, 4, 6, 7, 16, 17] and decreased use of outpatient care [3–5]. This study builds on this previous literature and strengthens the findings through the use of random voucher selection which can control for unmeasured differences between treatment and control groups that might otherwise bias our results.

The observed impacts of obtaining housing on health care in this study are aligned with the health systems transformation goal of improving health and health care quality. This suggests that meeting the housing sector’s goal of increasing housing access and stability also supports the goals of the health care sector’s health transformation efforts. In fact, this alignment in goals across sectors has already been recognized and has led to a number of partnerships and investments: for example, the Center for Medicare and Medicaid Services allows Medicaid dollars to be spent on housing services and resources, and some health care and hospital systems have even invested in brick and mortar housing [18]. Thus, cross-sector partnerships between health care and housing have the potential to improve health more than a single sector addressing it alone.

This study also provides evidence of how local HCV distribution policy can have cross-sector impacts. Importantly, the impact on health care utilization varied depending on the population prioritized by the local distribution policy. Housing Authority A’s distribution policy prioritized seniors/people with disabilities and individuals engaged in an education and employment training program while Housing Authority B prioritized families with school-aged children and medically complex individuals. This led to different demographics receiving the vouchers, specifically differences in age groups: Housing Authority B’s population was more likely to be concentrated in the 25–45 year age range, probably because this age range is more likely to have school-aged children. Typically, younger populations tend to have less need for outpatient services outside of their standard preventative care while older populations often require more outpatient care. Furthermore, our data shows that the increase in outpatient care for the Housing Authority A population was driven by specialty care, suggesting a greater need for care to manage health conditions often seen in older populations. Thus, the differences in outpatient care between the two agencies are likely explained, at least in part, by the target population served.

Another aspect of the voucher policy implementation that can impact outcomes is the definition of homelessness used to determine eligibility. At Housing Authority B, to be considered homeless and therefore eligible for an voucher, the applicant must be living in a shelter, car, or on the street. This definition of homelessness was not applied in the Housing Authority A prioritization strategy or eligibility criteria. Thus, it is possible that more of the Housing Authority B population who received a voucher were coming directly from the street, shelter, or car than the Housing Authority A population (however, we do not have prior housing data to confirm), meaning that Housing Authority B’s prioritization policy may explicitly target a population that is known to experience substantial barriers to accessing health care. Previous research has shown that homeless individuals living on the street are more likely to use the emergency department [16]; it may be that this requirement of homelessness in the Housing Authority B population is driving the observed difference in ED utilization between Housing Authority B treatment and control groups.

Finally, there are other factors that may have contributed to the differences in the observed outcomes. First, the prioritization policies may unintentionally lead to differences in population demographics, such as the greater proportion of female applicants in Housing Authority B’s population (also potentially due to their prioritization for homeless families with school-aged children). Second, there were differences in race and ethnicity in the study population across the two housing authorities. This likely stemmed from differences in the demographic make-up of the populations served by each housing authority and this could have impacted the use of healthcare. Finally, previous research has suggested that obtaining housing is associated with increased health care coverage, which in turn can result in increased outpatient care to address unmet health needs [19]. However, our analysis is limited to only those with Medicaid, so this was not as relevant in our analysis specifically but should be considered when examining policy implications.

The majority of Public Housing Authorities employ preferences to prioritize certain populations for their vouchers [13]. Previous studies have explored the differential impacts of different types of housing assistance on health outcomes,[20] as well as the different health and social needs of individuals housed through different programs [21]; however, to our knowledge, no other studies have explored the impact different prioritization policies have on the health care outcomes of individuals housed through the HCV program. As long as demand for vouchers continues to far outweigh supply, housing authorities will need to ration vouchers. The impact of these prioritization strategies on health care suggests that these policies can inform local partnerships between housing and health care agencies and could be part of a larger strategy to improve community health.

There are limitations to consider when interpreting the findings of this study. The study population was limited to individuals on Medicaid; it may not be generalizable to individuals with other coverage or the uninsured. Furthermore, both housing agencies are located in majority White counties; findings may not reflect the experiences of more diverse populations. The study also has a relatively small sample size, which may have limited our ability to observe statistical significance. It is possible, then, that the small sample size yielded non-significant results, even when there was an effect of housing on a specific health care domain.

While the study provides important evidence for the associations between housing and health care, and the way in which local policy can impact that association, we were unable to assess the full impacts of the voucher. Because we were only able to assess the impact of utilization on applicants, we cannot conclude if the voucher had an impact on household members. In particular, this is a limitation among the Housing Authority B population, most of whom had children present in their household. Children have substantially different expected health care utilization patterns than adults, and future studies should explore the impact of HCV prioritization strategies on children’s health care outcomes. Likewise, we had to exclude adults over the age of 65 years as we did not have access to Medicare claims. Seniors also have a substantially different expected health care utilization pattern, compared to younger adults, and the exclusion of these adults who are more prevalent in the Housing Authority A population may have masked even greater differences in impact between the two distribution policies. Thus future studies should obtain waitlist data on the entire household if possible, in order to more comprehensively understand the impact of the HCV program.

Additionally, we were unable to assess the impact of obtaining housing through the HCV program on other sectors. Previous research has demonstrated the broad impact housing can have on individual and community health and wellbeing, including impacts on children’s education, household food and nutrition access, and interactions with the criminal legal system [22–24]. Given the interconnectedness of these sectors, future research should bring together multiple data sources to more fully understand the long-term impact of housing assistance.

This study provides evidence that, while obtaining housing through the HCV program can result in better health care outcomes overall, local prioritization policies can influence that impact. The evidence demonstrates the cross-sector impact of HCV policy and provides further data for the importance of close connection and collaboration between housing and health care.
